# A Minimally Invasive Treatment Approach for Early-Stage Uterine Cervical Cancer: The Impact of the LACC Trial and a Literature Review

**DOI:** 10.3390/medicina61040620

**Published:** 2025-03-28

**Authors:** Elena-Mihaela Vrabie, Mihai-Adrian Eftimie, Irina Balescu, Camelia Diaconu, Nicolae Bacalbasa

**Affiliations:** 1 Department of Visceral Surgery, Center of Excellence in Translational Medicine, Fundeni Clinical Institute, 022328 Bucharest, Romania; elena.vrabie@drd.umfcd.ro (E.-M.V.); mihai.eftimie@umfcd.ro (M.-A.E.); nicolae.bacalbasa@umfcd.ro (N.B.); 2 Department of Surgery, “Carol Davila” University of Medicine and Pharmacy, 050471 Bucharest, Romania; 3 Department of Internal Medicine, Floreasca Clinical Emergency Hospital, 030084 Bucharest, Romania; camelia.diaconu@umfcd.ro; 4 Department of Internal Medicine, “Carol Davila” University of Medicine and Pharmacy, 050471 Bucharest, Romania

**Keywords:** uterine cervical cancer, laparoscopy, robotic surgery

## Abstract

*Background and Objectives:* Recent studies have supported the non-inferiority of the minimally invasive treatment approach over the open approach. However, they have also underlined its inferiority regarding its oncological results, while preserving the short-term benefits. The direct effects of these results were represented by indication changes in international guidelines on the application of minimally invasive surgery for treating early-stage cervical cancer. *Material and metods:* Herein, a literature review, including studies between 1992 and 2017, was performed. *Results:* The results show that the studies published during this period supported the non-inferiority of the minimally invasive treatment approach for early-stage cervical cancer compared with the open approach. However, the studies included were unicentric, non-randomized and relied on a reduced number of patients. The results of the Laparoscopic Approach to Cervical Cancer [LACC] trial could not have been considered, since only studies published between 1992 and 2017 were included. This trial firmly supported the advantages of the minimally invasive approach in treating early-stage cervical cancer. The literature published after 2018 highlighted the necessity for new clinical studies, randomized and prospective ones, to cover the defects of this study and to verify (or not) its results. *Conclusions*: the studies published after 2018 mainly focused on the deficiencies of the LACC trial and also on developing new methods that could improve this surgical technique, thus enhancing the safety of the minimally invasive approach in treating early-stage cervical cancer. However, none of the included studies succeeded to provide enough evidence to oppose the results obtained in the LACC trial. Therefore, in order to clarify the state of this surgical approach, the results of three ongoing randomized clinical trials are expected.

## 1. Introduction

Uterine cervical cancer, a significant public health issue, is the fourth most common type of cancer in women worldwide, with an incidence and mortality of 604,000 and 342,000 cases, respectively, according to the World Health Organization 2020 [1]. The most significant and recognized cause associated with the development of cervical cancer is persistent infection with human papilloma virus (HPV). Recently, the prognosis of this disease has been improved due to the introduction of screening programs for HPV infection, and more particularly for oncogenic types 16 and 18, thus allowing the early detection and treatment of premalignant lesions. In addition, it has been reported that the increase in vaccination rates against HPV infection can prevent the onset of ~70% of cervical cancer cases.

The treatment of cervical cancer is complex and commonly includes radical surgery, systemic chemotherapy, brachytherapy and radiotherapy. In addition, a multidisciplinary team is required for the treatment of the disease. In the early stages of the disease International Federation of Gynecology and Obstetrics ((FIGO) stage IA1, without lymphovascular invasion), conservative treatment with cervical conization could be considered, since this stage is characterized by a low incidence of lymphatic spread. Additionally, conservative surgery can also be performed in stages IA2 and IB1 in well-selected patients, when the tumor diameter is <2 cm. In stages IA2, IB1 and IIA1, optimal surgical treatment with total radical hysterectomy with bilateral salpingo-oophorectomy and pelvic lymph node dissection is recommended. In more advanced stages, beyond IIA2, definitive systemic chemotherapy is preferred to radical surgery. Pelvic exenteration is a potential option for recurrent or persistent cervical cancer without distant metastases, while in locally advanced disease or in patients who are not candidates for surgical treatment, radiotherapy is a reliable option. According to the latest treatment guidelines, laparotomy is considered as the standard surgical approach. However, the minimally invasive approach (laparoscopic or robotic) is still valid in well-selected patients [1,2].

The current study examined the evolution of the surgical approach in the treatment of cervical cancer, from the introduction of the minimally invasive methods to their improvement and increased use, until the publication of the study by Ramirez et al. [3] in 2018. The above study made scientists and physicians doubt the oncological safety of this surgical approach and aimed to modify the trends in the following years [4,5,6,7,8,9,10,11,12,13,14,15,16,17,18,19].

## 2. Materials and Methods

**Figure 1:**
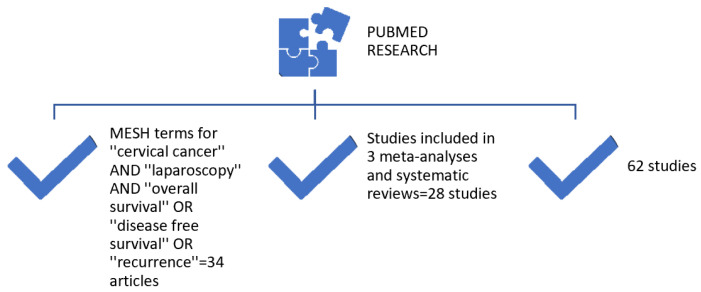
Methodology.

A search in only PubMed was conducted (without using other databases) between 1992 and 2017 using the MESH terms “cervical cancer” (“cervical cancer” or “uterine cancer” or “uterine cervical cancer”) AND “laparoscopy” OR “robotic surgery” (“laparoscopy” or “laparoscopic surgery” or “laparoscopic-assisted surgery” or “robotic surgery” or “robotic-assisted surgery”) AND “overall survival” OR “disease-free survival” OR “recurrence” (“overall survival” or “disease-free survival” or “recurrence” or “relapse”). Considering that the application of the minimally invasive approach in the treatment of uterine cervical cancer began in 1992, the search began in this year and ended in 2017, since the first results of the Laparoscopic Approach to Cervical Cancer (LACC) trial were published in early 2018. This trial changed the perspective of this surgical approach. Additionally, only studies in English or Romanian were included, while in terms of article type, conference proceedings, clinical trials, clinical studies, comparative studies, controlled clinical trials, randomized clinical trials, pragmatic clinical trials, evaluation studies and multicenter studies were screened (Table 1). A total of 158 articles were retrieved. The exclusion criteria were as follows: studies with incomplete data (no data regarding relapse rate or disease-free survival); studies including patients with stages beyond IIB; those with patients who underwent fertility preservation surgery; and meta-analyses or systematic reviews that were not excluded by selecting the previous additional criteria (Table 2). Therefore, finally, a total of 34 articles were included in the current article. All articles were analyzed and read in full text. Subsequently, three meta-analyses and systematic reviews—published between 1992 and 2017, on the effect of the minimally invasive approach on cervical cancer in terms of survival, disease-free survival and recurrence rates—were reviewed, namely those by Cao et al. [20], Wang et al. [21] and Park et al. [22]. Additionally, the studies extracted from the above meta-analyses and systematic reviews [*n* = 28] were also evaluated (Figure 1). Therefore, a total of 62 studies were reviewed (Table 3). Among all studies included, 26 were prospective ones. In terms of surgery type, 31 studies were on the application of the total laparoscopic approach [total radical hysterectomy using total laparoscopic approach], 10 on the robotic approach and 13 on laparoscopically assisted transvaginal total radical hysterectomy. Furthermore, a total of 46 comparative studies were included in the current review article, with 8 comparing the open approach with laparoscopic-assisted transvaginal total radical hysterectomy, 20 open approach with total laparoscopic approach, 7 open approach with robotic approach and 8 open approach with robotic and total laparoscopic approaches. Finally, studies published after 2018 were also reviewed, and the most significant findings and comments of the LACC trial were noted (Table 4).

## 3. Results

Herein, the exploration of the minimally invasive approach in the treatment of early-stage uterine cervical cancer between 1992 and 2018 was based on literature reports, which strongly suggested that its results were comparable to those obtained by laparotomy. However, the majority of these studies were retrospective (56%) or non-randomized, prospective or unicentric.

Starting from 2002, Spirtos et al. [23] supported the effectiveness of laparoscopic total radical hysterectomy via studying a cohort of 78 patients with stage IA2 and IB cervical cancer. Therefore, a 5-year survival rate of 93.6% and a 5-year disease-free survival rate of 89.7% were reported, with a recurrence rate of 10.3% at 66.8 months after surgery. These results were comparable with those obtained by Delgado et al. [24,25], who studied a group of 645 patients with stage I cervical cancer treated by the open approach, with a 3-year disease-free survival rate of 94.8% in cases where the tumor diameter was >3 mm and 58.5% in cases where the tumor diameter was >21 mm. In 2003, Hertel et al. [24] prospectively evaluated a cohort of 200 patients who underwent laparoscopically assisted total radical transvaginal hysterectomy. A 5-year survival rate of 83% and a recurrence rate of 18.5% were recorded, with an estimated 5-year survival of 98% after correction for independent prognostic factors. Consequently, this approach was considered ideal in cases where the tumor was <4 cm in diameter, negative for lymph nodes and without lymphovascular and angiovascular invasion. This finding was also supported by the studies by Steed et al. [26], Jackson et al. [27] and those published after 2010 [28,29,30,31,32,33].

Later, Pomel et al. [34] published a retrospective study of 50 patients with stage IA2 and IB1 cervical cancer who underwent laparoscopic surgery, with a median follow-up of 44 months and a 5-year survival rate of 96%, thus verifying that this treatment strategy could not compromise survival.

Additionally, in 2005, Moreno et al. [35] retrospectively followed a group of 27 patients with stage IA2 and IB1 cervical cancer who underwent laparoscopic total radical hysterectomy. The survival rate within a median follow-up period of 32 months was 100%, thus further supporting the safety and feasibility of this method as an alternative to open surgery.

In another study in 2007, the authors compared the recurrence and mortality rates in patients with stage IB–IIA cervical cancer who underwent laparoscopic total radical hysterectomy with pelvic lymph node dissection versus open surgery. The analysis highlighted the oncologic safety and efficacy of the minimally invasive approach, when the surgery was performed by experienced surgeons [36]. This finding was further supported in 2008 by Pellegrino et al. [37] and other prospective longitudinal studies published in 2010 [38,39,40,41,42,43]. Furthermore, the oncologic safety of laparoscopic total radical hysterectomy with pelvic lymph node dissection was also confirmed in the observational study published by Xiao et al. [44,45,46]. This study followed a total of 154 patients over a period of 13 years. The same encouraging results were obtained by Yang et al. [47], further indicating that laparoscopic surgery was an adequate, safe and feasible approach that could be applied as a primary treatment method for early-stage cervical cancer.

In 2012, Park et al. [48] retrospectively evaluated the effect of minimally invasive surgery on treating patients of >65 years of age with stage IB–IIA cervical cancer. The results demonstrated that this approach was associated with fewer postoperative complications, without reducing 5-year disease-free survival or overall survival.

Meanwhile, Nam et al. [49] compared the overall survival and recurrence rates between patients who underwent open surgery and those treated with laparoscopic surgery. Therefore, no differences were observed, even in cases with tumors >2 cm in diameter. Consistently, in 2014, another retrospective study verified that laparoscopic surgery was feasible in 88 patients with stage IB–IIA cervical cancer and tumor diameter of >3 cm. In addition, no notable differences in terms of disease-free survival were observed compared with laparotomy [50].

A previous propensity-matched prospective study, including 130 patients with cervical cancer, evaluated the long-term results of laparoscopic surgery compared with the open approach. The results showed a similar 5-year disease-free survival rate between the two groups [51,52,53,54]. Additionally, Hoogendam et al. [55] monitored 100 patients with early-stage cervical cancer who underwent radical surgery using the robotic approach at a tertiary center. The results of this observational cohort study reported a 5-year disease-free survival rate and overall survival rate of 81.4 and 88.7%, respectively. The data for the open approach were comparable with those prevailing at the time, thus reassuring the role of minimally invasive surgery.

In addition, other studies published in 2015 supported that the laparoscopic approach was a safe and feasible alternative to open surgery, when performed by experienced surgeons [56,57,58,59,60,61,62,63].

In 2016, Kong et al. [64] analyzed the recurrence pattern of patients who underwent laparoscopic or robotic total radical hysterectomy with intracorporeal colpotomy versus vaginal colpotomy. Therefore, a higher recurrence rate was observed in patients who were treated with intracorporeal colpotomy. This finding could be due to the CO_2_ pneumoperitoneum-triggered intraperitoneal dissemination of tumor cells and the higher rates of vaginal positive margins in this type of colpotomy. The data obtained in this study could be of particular interest in the coming years as part of the possible explanation for the poor oncological results of minimally invasive surgery.

Regardless of the results of the previously reported studies, a retrospective study by Laterza et al. [65] reported similar recurrence rates between patients with early-stage cervical cancer who underwent laparoscopic surgery compared with those who were treated with the open approach. In 2017, a retrospective cohort study, including 383 patients from two medical reference centers, compared the minimally invasive approach [both laparoscopic and robotic] with the open approach. The analysis revealed similar recurrence rates between the two approaches, while a higher number of harvested lymph nodes was recorded in the minimally invasive approach. However, no differences in the number of specimen positive margins and adjuvant oncological treatment rates were observed [66].

A meta-analysis published by Cao et al. [36,38,50,51,56,67,68,69,70,71,72,73,74,75,76,77,78,79] in 2015, including 2922 cases of cervical cancer, confirmed that laparoscopic total radical hysterectomy was oncologically safe, with no notable differences in overall survival and prognosis compared with open surgery. Similarly, another meta-analysis, performed on a set of 12 original studies, indicated that the 5-year oncological outcomes were similar between the laparoscopic and open approaches [36,49,51,56,69,70,71,72,77,79,80,81]. In 2016, a meta-analysis composed of 15 studies compared the laparoscopic approach with the robotic and open approaches. The results showed that there were no differences in overall survival between these techniques [82,83,84,85,86,87,88,89,90,91,92,93,94,95,96,97]. However, more studies are needed to accurately determine the safety and efficacy of the robotic approach.

Finally, the literature published before 2018 (Table 1), including the aforementioned retrospective and non-randomized prospective studies, as well as other studies [98,99,100,101,102,103,104,105,106,107,108,109,110,111,112], suggested that the minimally invasive approach (laparoscopic or robotic) was oncologically safe and did not compromise disease-free and overall survival rates, while it did not affect recurrence rates. Despite brief evidence that intracorporeal colpotomy could increase recurrence rates, systematic vaginal colpotomy was not implemented, since larger studies are needed to establish its indications.

Between 2008 and 2017, Ramirez et al. [3] conducted a multicenter, randomized, prospective study analyzing the 3-year survival, 4.5-year disease-free survival and recurrence rates in patients with stages IA1, IA2 and IB1 cervical cancer, who underwent minimally invasive (84.4% and 15.6%, laparoscopic and robotic surgery, respectively) or open surgery. The results of this study were alarming. A higher recurrence rate was recorded in the minimally invasive surgery group compared with the open surgery group (8.4% versus 2.2%). In addition, the 4.5-year disease-free survival rate was reduced to 86% in minimally invasive surgery compared with 96.5% in open surgery and the 3-year survival rate to 93.8% from 99%, respectively. However, due to oncologic safety concerns, the study was stopped early.

## 4. Discussion

Despite several improvements in screening, diagnosis and treatment strategies, cervical cancer remains an important health problem, with different patterns of recurrence affecting oncologic outcomes, even in early-stage disease.

Recurrence after primary treatment for cervical cancer most commonly occurs at the vaginal vault alone, cervix, uterus, parametria [local recurrence] and surrounding organs or pelvic lymph nodes (regional recurrence). However, distant metastasis in infra- or supra-diaphragmatic lymph nodes and distant organs is considered as the predominant pattern of relapse in locally advanced cervical cancer [113,114,115,116]. The pattern of recurrence serves as a significant predictor of cervical cancer prognosis. A study demonstrated that patients with distant lymph node and peritoneal recurrence had better outcomes compared with those with other recurrence sites. The latter needed more aggressive therapeutic strategies to improve survival [117]. It seems that local recurrence is an event seen in high-risk patients with worse outcomes compared with distant recurrence. Given the frequent relapse in the paravaginal tissue containing the pelvic plexus, more studies are needed to evaluate the role of nerve-sparing surgery and the involvement of perineural invasion in early-stage cervical cancer [118].

Nodal involvement can also negatively affect the prognosis of patients with cervical cancer. Emerging evidence has suggested that there is a difference in time to recurrence and overall survival between patients of stage cN0 and cN1. However, the sites of recurrence remain the same. Although routine lymphadenectomy is associated with several postoperative complications, nodal involvement still may or may not be evaluated in a patient-tailored scheme. The study by Ji et al. [119] underlined the need for ’a precise definition of the risk of lymph node metastasis in early-stage cervical cancer’ to establish an indication for lymphadenectomy. Additionally, the study stated that the minimally invasive approach could be useful for exploring the localized lymph node recurrences. The risk of recurrent disease is higher in the first few years after treatment. However, there is no consensus on the follow-up of these patients, with several studies suggesting a patient-specific surveillance and treatment plan.

Unscheduled follow-up is required if the patient develops symptoms, such as abdominal or pelvic pain, vaginal bleeding or urinary dysfunction. An increase in tumor marker levels, such as those of cancer antigen 125 and SCC or squamous cell carcinoma antigen, is commonly associated with disease recurrence and therefore follow-up should be initiated [113,120,121]. According to the National Comprehensive Cancer Network (NCCN) guidelines, if the patient remains asymptomatic, evaluation should be performed every 3–6 months for the first 2 years and then every 6 months for the next 3 years. The surveillance imaging techniques are represented by magnetic resonance imaging (MRI) scan, computed tomography (CT) scan and fluorine-18 fluorodeoxyglucose (18F-FDG) positron emission tomography (PET)/CT. MRI scanning is used to assess local recurrence and adjacent organ invasion, while CT and 18F-FDG PET/CT scans are used to detect distant metastases [116].

After the publication of the study by Ramirez et al. [3], several studies have evaluated whether recurrence rates are affected by the surgical approach in early-stage cervical cancer. In 2023, Corrado et al. [119,122] published the results of a study evaluating the patterns of recurrence in 360 patients with FIGO stage IB1–IB2 cervical cancer treated with laparotomy or minimally invasive surgery [laparoscopic or robotic]. The analysis showed a recurrence rate of 16.4%, with no differences in the recurrence patterns between the open and minimally invasive groups. Additionally, no statistically significant differences were observed in disease-free survival and overall survival between the two groups. The above results contradicted those reported in a systematic review and meta-analysis by Nitecki et al. [123], which evaluated the risk of recurrence after minimally invasive surgery compared with open surgery. The results demonstrated that the minimally invasive surgery was associated with an increased risk of recurrence. At the same time, the study published by Sert et al. [124] in 2021 found that minimally invasive radical hysterectomy was associated with a reduced time to recurrence and reduced overall survival compared with open radical hysterectomy. A high risk for peritoneal recurrence was reported, even for tumors of <2 cm in diameter. The above findings were inconsistent with those obtained by Corrado et al. [122]. Regarding the patterns of recurrence observed in patients treated by the minimally invasive approach, the study by Fitzsimmons et al. [125] revealed that the application of the robotic approach for tumors of >2 cm was associated with carcinomatosis (45 % of all recurrences) and reduced overall survival.

Levine et al. [126] suggested that the different outcomes between minimally invasive and open surgery regarding disease-free and overall survival in patients with early-stage cervical cancer could be due to the differences in adopting the international guideline indications for adjuvant therapy.

In 2024, Ramirez et al. [127] published a study entitled “Final Analysis on Overall Survival Comparing Open Versus Minimally Invasive Radical Hysterectomy for Early-Stage Cervical Cancer” and stated that “given the higher recurrence rate and worse overall survival with minimally invasive surgery, an open approach should be the standard of care”. Furthermore, the impact of the initial results of the Ramirez et al. [3] study was really important and led to changes in the national and international guidelines regarding the application of the surgical approach in the treatment of cervical cancer.

The NCCN guidelines (version, 3.2019) suggested that the patients should be informed regarding the study’s results, but also about the short-term benefits of the minimally invasive approach [128]. The newest version (1.2023) affirmed that laparotomy should be considered as the standard surgical approach in treating uterine cervical cancer [129]. Additionally, the European Society of Medical Oncology (ESMO) guidelines, updated in 2020, reported that in stages IA2, IB and IIA, laparoscopic or robotic total radical hysterectomy should not be considered as the appropriate method of treatment compared with laparotomy, while the patients should be counseled regarding the risks and benefits of the laparoscopic approach [2]. Furthermore, the European Society of Gynaecological Oncology [ESGO]/European Society for Radiotherapy and Oncology (ESTRO)/European Society of Pathology (ESP) guidelines updated in 2023 indicated that the minimally invasive approach could be used in low-risk tumors of <2 cm diameter or with free margins after conization, in high volume centers and patients who had been previously informed about the study’s results [130]. Finally, the MD Anderson Cancer Center also suggested that robotic total radical hysterectomy could be an option for some selected patients [131]. Although international guidelines and much of the current literature indicated that the open approach should be considered as the standard of care for cervical cancer, there are still studies that support the application of minimally invasive surgery if some improvements in surgical technique are adopted to prevent tumor cell spillage via exposing the tumor by a uterine manipulator or via directly manipulating the cervix. These modifications include avoiding the use of a uterine manipulator and complete vaginal transection during laparoscopy, due to the increased risk of intraperitoneal tumor cell dissemination [132]. Another study also suggested that protective colpotomy could result in similar recurrence-free and overall survival rates compared with abdominal radical hysterectomy [133]. The above protective measures against tumor cell spillage, combined with better control of the surgeon’s learning curve, could avoid the abandonment of the minimally invasive approach for treating cervical cancer, thus leading to equivalent oncologic outcomes compared with open surgery [131,132,134,135,136]. However, in the study by Li et al. [137], minimally invasive surgery without the use of a uterine manipulator was still inferior in treating early-stage cervical cancer in terms of disease-free survival compared with open surgery.

The results of the LACC trial caused several reactions in the medical literature. In 2018, Kimming et al. [138] commented on the consequences of this publication and raised the problem of the insufficient learning curve of the participating surgeons. Therefore, in the LACC trial, the surgeons should only have the experience of at least 10 laparoscopic cases and submitted two unedited videos, thus resulting in ~2 patients treated per year per center. However, this was considered insufficient to maintain training and experience. It was also stated that the results of the study could be imprecise due to the incomplete data presentation, the lack of histopathological data regarding tumor diameter in 1/3 of the cases and the lack of information regarding lymphovascular invasion in 5% of the cases, as well as regarding vaginal or parametrial invasion in 7–10% of the cases. Furthermore, Hillemanns et al. [139] questioned the accuracy of the trial results considering the small number of participants, the incomplete data presentation and the lack of a standardized learning curve. The issue of the learning curve of surgeons and the lack of standardized techniques was considered as a factor that could negatively affect the results of the LACC trial. The surgeon’s expertise is recognized as being highly associated with oncologic outcomes, not only in the minimally invasive surgery of uterine cervical cancer, but also in minimally invasive surgery for colorectal cancer [140,141]. Previous studies found that when the surgeon was in the early phase of the learning curve, the prognosis was poorer compared with those in the late phase and, therefore, there should be a minimum number of cases required to achieve surgical proficiency [142]. Also, J. H. Ahn conducted a retrospective study assessing the disease-free survival in two groups—group 1 was treated by surgeons in the beginning of the learning curve and group 2 was treated by experienced laparoscopic surgeons—and concluded that the relapse rate was higher in group 1 and that the only predictor for the poor disease-free survival in this group was the surgical proficiency [143]. H. Falconer stated that “the learning curve should be considered institutional’’, meaning that the more experienced surgeons could represent an important support for the ones newly initiated in the laparoscopic approach and may improve the learning process [144]. Moreover, G. Moufawad emphasizes that the lack of training in laparoscopic surgery in order to perform radical hysterectomy for cervical cancer should be addressed and special training programs should be implemented in order to avoid the complications and the negative impact on the oncological outcomes [145]. The criticism against the insufficient learning curve of the surgeons involved in the LACC trial was further justified taking into account that in several studies, the risk of recurrence only dropped after 19 surgeons respectively operated on 61 melanoma in situ cases, numbers well above those accepted in the LACC trial [146,147]. In addition, other data supported that when the surgeon was more experienced, there were no oncological differences between the open and robotic approach [148]. Other studies compared the overall survival and disease-free survival observed in different phases of the learning curve and found that the results were comparable with those from the LACC trial, when the surgeons were in the early phase of the learning curve [149]. Based on the aforementioned findings, the surgeon’s proficiency could have been compromised in the LACC trial and it is therefore necessary to acknowledge this when analyzing the study’s results.

Regarding the surgical technique, a previous study supported that the use of a uterine manipulator could favor tumor spillage and elevate the recurrence risk in patients with tumors of >2 cm in diameter, while also reducing overall survival [150]. Interestingly, other studies showed that when the uterine manipulator was no longer used, the recurrence risk remained still higher or comparable with open surgery [137,150]. These results suggested that other factors could be associated with the negative outcomes of the surgical technique, such as unprotected colpotomy. Additionally, protective vaginal closure could display similar recurrence rates to those observed in the open surgery groups [150,151]. Nevertheless, the histopathological data (positive margins or parametria, lymph node metastasis, presence of residual tumor at hysterectomy and tumor size) could play an extremely significant role, taking into account that after controlling for these factors, the use of a uterine manipulator or unprotected colpotomy were no longer associated with worse outcomes [152]. Tumor size is strongly associated with lymph node metastasis and parametrial involvement rates. Therefore, since tumors <2 cm in diameter are considered low risk, the patients could not benefit from radical surgery. Another study also indicated that minimally invasive surgery was not associated with worse oncological outcomes compared with open surgery in tumors of <2 cm in diameter [146]. The LACC trial suggested that tumor size was equally balanced between groups. However, the results could not be generalized to low-risk tumors. Therefore, the results of randomized studies on the appropriate surgical approach for low-risk tumors are awaited [153].

The LACC trial could not provide a standardized learning curve, surgical technique and histopathological evaluation. Therefore, its results should definitely be re-analyzed in light of recent data.

In light of the results of the LACC trial, Liang et al. [154] aimed to answer whether the minimally invasive approach could be used to treat cervical cancer. This study supported that the negative results of the LACC trial could be due to complete vaginal transection during the laparoscopic time, which could eventually lead to an increased risk of intraperitoneal tumor cell dissemination. Therefore, vaginal total radical hysterectomy with bilateral salpingo-oophorectomy completed by laparoscopic lymph node dissection could be performed. The poor results of the LACC trial could also be caused by the deficient selection of surgeons. Therefore, more complete results could only be obtained in another randomized prospective study, carried out by a single pathologist, with standardized preoperative MRI and parametrial measurements, as well as with quality indicators regarding radical hysterectomy and a larger number of patients. Bogani et al. [155] supported the idea of another randomized prospective study, which should cover the insufficiencies of the LACC study and the implementation of the methods designed to prevent intraperitoneal contamination.

The international guideline updates after 2018 indicate that the minimally invasive approach should not be abandoned yet. However, the patient selection system should be improved, while given the encouraging results of minimally invasive surgery in tumors of <2 cm in diameter, the perioperative MRI and tumor size measurements should be standardized. Additionally, since different histologic types could have different prognoses, clearer histopathologic results should also be obtained.

To better understand and establish patient-specific treatment strategies, the recent literature has also focused on the role of different histologic types of cervical cancer as prognostic factors. Cervical adenocarcinoma and squamous cell carcinoma are the most common types of cervical cancer and are known to have a similar treatment response and prognosis. It has been reported that patients with adenocarcinoma exhibit lower overall survival compared with those with squamous cell carcinoma, with adenocarcinoma being considered as an independent prognostic factor for poor survival and high risk of distant recurrence in early-stage disease [156,157,158]. Regarding the surgical approach, the study by Giannini et al. [159] did not reveal any significant differences between open surgery and minimally invasive surgery in patients with FIGO IB–IIA cervical adenocarcinoma, thus supporting the significant role of minimally invasive surgery in treating cervical carcinoma regardless the histologic type. Mucinous adenocarcinoma is less common than the usual type or gastric type [160]. However, this type is characterized by enhanced invasiveness and difficulty in early diagnosis. The study by Hao et al. [161] established a prognostic model for mucinous adenocarcinoma, which could assist the identification of relevant risk factors, implementation of appropriate therapy and outcome prognosis. The nomogram developed in this study proved to be more accurate in predicting 1-, 3- and 5-year survival in patients with mucinous adenocarcinoma compared with the American Joint Committee on Cancer and FIGO stages.

A previous retrospective study evaluated the use of the laparoscopic approach before and after the publication of the LACC trial results, particularly between November 2015 and March 2020. Therefore, prior to the LACC trial, the minimally invasive approach was applied in 58% of cervical cancer cases, and decreased to 42.9% after the study was published. Additionally, the likelihood of choosing laparoscopy for the treatment of uterine cervical cancer decreased to 59% after the study was published [162].

A potentially safe alternative to laparoscopic radical hysterectomy in early-stage cervical cancer is represented by laparo-assisted vaginal radical hysterectomy. The study conducted by C. Ronsini in 2022 reported that this surgical technique provides comparable disease-free survival and overall survival with the open approach and that it deserves to be learned and practiced as a viable option [163]. Also, the use of laparoscopic-assisted vaginal radical hysterectomy was supported by the meta-analysis published by Zeng 2022 [164] which underlined its advantages compared to the open approach: decreased blood loss, shorter hospitalization, fewer urinary tract infections and potentially a lower relapse rate [164]. Taking into account that as a result of the LACC trial the use of laparoscopic radical hysterectomy seemed to be prohibited, the laparoscopic-assisted vaginal radical hysterectomy might represent the optimal alternative in treating early-stage cervical cancer.

There are currently three ongoing randomized prospective phase 3 trials, namely that of Chao et al. [165], evaluating the application of laparoscopy and robotic surgery compared with laparotomy alone for the treatment of uterine cervical cancer, that of Falconer et al. [166] comparing the robotic approach with laparotomy and that of Wu et al. [167] comparing laparoscopy with laparotomy.

The study conducted by Chao has a primary objective to assess the 5-year disease-free survival in patients with early-stage cervical cancer treated by the open approach compared to the minimally invasive approach and secondary objectives to determine the 5-year overall survival, to analyze the impact of different surgical approaches on the quality of life and pelvic floor dysfunction, to discuss the economic aspects related to each surgical approach, to clarify the correlation between the use of uterine manipulators and the risk of relapse, and to explore the specific recurrence sites in the both minimally invasive and open approaches. The strength of this study is expected to be represented by the emphasis on “surgeons as one of the important parameters for the survival analysis’’ [165].

The study conducted by Falconer aims to prove the hypothesis that robotic-assisted radical hysterectomy has non-inferior recurrence-free survival compared to the open approach and that it improves the patient’s quality of life. Also, it will investigate the role of sentinel lymph node biopsy using indocyanine green as a diagnosis tool in order to replace the systematic lymph node dissection in women with early-stage cervical cancer [166].

Eventually, the study conducted by Wu will firstly assess the 5-year disease-free survival in laparoscopic versus open radical hysterectomy as well as the secondary survival rate, relapse rate and perioperative results related to each surgical approach [167].

The results of the aforementioned trials are expected to clarify, to some extent, the effectiveness of the surgical approach in the treatment of cervical cancer.

The most recent published studies confirm that the results of the LACC trial were not the final conclusion regarding the state of minimally invasive surgery in treating early-stage cervical cancer and that the surgical technique review along with the advancements in tumor spillage prevention during laparoscopy are effective in supporting the use of this surgical approach in well-experienced centers [168]. While there is still doubt and the results of the ongoing trials are still pending, the recent literature and guidelines recommend the use of minimally invasive surgery in early-stage low-risk cervical cancer with tumor diameter < 2 cm and negative margins after conization [169,170,171,172,173,174,175,176].

In January 2025, P. T. Ramirez published an editorial discussing the latest studies regarding the state of minimally invasive surgery in early-stage cervical cancer. The results of the SHAPE trial were mentioned with emphasis on the oncologic safety of the minimally invasive approach for simple hysterectomy in patients with low-risk cervical cancer. The low-risk cases were defined as “squamous carcinoma, adenocarcinoma, or adenosquamous carcinoma, FIGO 2009 stage IA2 or IB1 tumors with lesions measuring no more than 2 cm, limited depth of invasion (<10 mm), MRI showing <50% invasion, any histologic grade, and no evidence of lymph node metastases’’. The study supported the fact that simple hysterectomy with lymph node evaluation was not inferior to radical hysterectomy in patients matching the criteria listed before in terms of 3-year pelvic recurrence [177,178].

Also in January 2025, M. Plante analyzed a group of patients from the SHAPE trial who underwent minimally invasive simple hysterectomy versus open simple hysterectomy and concluded that the outcomes did not differ between the two approaches in terms of recurrence rates (4.3% in minimally invasive group and 5,3% in open group) [179].

## 5. Conclusions

Finally, the role of minimally invasive surgery in the treatment of cervical cancer still remains controversial, since the previously published studies could not establish the oncological safety of this treatment approach. Therefore, further studies are needed to clearly define its status. The LACC trial results across all minimally invasive surgeries could not be generalized, taking into account the established role of these types of surgery in colorectal cancer, body and pancreatic tail cancer, liver metastases and endometrial cancer. As already discussed, the surgeon’s expertise, patient selection (at least based on tumor size), distinguishing low- and high-risk tumors, and improving surgical techniques via using protective measures are extremely significant for saving the role of minimally invasive surgery in the treatment of early uterine cervical cancer. As the results of recent randomized studies on this topic are awaited, there is hope that these milestones could be conquered, and the role of this surgical method could be precisely established.

The strengths of our study are represented by the following: the rigorous extended search and selection of the articles from the PubMed database discussing the literature published before the LACC trial and also after its publication; the emphasis on the multiple controversies around the main subject, the authors’ conclusions and the information provided regarding the patient selection for minimally invasive approach; and the discussion of methods to improve the surgical technique based on the latest published literature and national/international guideline updates.

The limitations of our study are represented by the use of only PubMed database, which might have led to the omission of relevant literature, and by the lack of new results from the ongoing randomized clinical trials. The follow-up of this paper should probably summarize the literature before 2018 and focus on the updates recently published regarding patient selection, surgeon training in the minimally invasive approach, and methods to improve the surgical technique and to increase the oncologic safety of this approach, complemented by the results of the randomized trials.

We strongly believe that our research is relevant in clinical practice because it provides a wide perspective about the evolution of the minimally invasive approach in treating early-stage cervical cancer, as well as reviews important aspects related to patient selection, surgical technique improvement, alternative options and the importance of surgeon specialized training helping the clinician to see the whole picture when establishing the therapeutic plan for a patient with early-stage cervical cancer. At the very least, the results of our research might contribute to the doctor–patient discussion when choosing the surgical approach based on relevant and validated literature.

## Figures and Tables

**Table 1:** Inclusion criteria.

Inclusion criteria	Year of publication 1992–2017
	PubMed database
	MESH terms for: - Cervical cancer - Laparoscopy - Robotic surgery - Overall survival - Disease-free survival - Recurrence
	English/Romanian language
	Article type: - Conference - Clinical trial - Clinical study - Comparative study - Controlled clinical trial - Randomized clinical trial - Evaluation study - Multicentric study

**Table 2:** Exclusion criteria.

Exclusion criteria	Incomplete data presentation
	Stages beyond IIB
	Use of fertility preservation surgery
	Meta-analyses or systematic reviews that were not excluded by selecting the previous additional criteria

**Table 3:** Literature published before 2018—main conclusions of these studies were cited.

First Author	Year of Publication	Main Conclusions
Hsieh YY	1998	- LRH is a safe procedure. - Short-term follow-up (1.3–5.1 years) with favorable prognosis.
Spirtos NM	2002	- LRH (type III) successfully completed in patients with early-stage cervical cancer with acceptable morbidity. - Intermediate-term follow-up validates the adequacy of this procedure.
Pomel C	2003	- Radical hysterectomy can be performed by laparoscopy in stage IB1 or less advanced node-negative cervical cancer patients without compromising survival. - Prior brachytherapy did not affect the feasibility of this radical procedure.
Steed H	2004	- LARVH has similar efficacy and recurrence rates to radical abdominal hysterectomy in early-stage cervical cancer patients.
Nam JH	2004	- LRH for the treatment of early cervical cancer is a safe and effective alternative to conventional radical hysterectomy. - LRH limited to patients with small volume disease (tumor diameter <2 cm or volume <4.2 cm). - Higher recurrence rate in patients with large tumor volume.
Gil-Moreno A	2005	- LRH can be successfully completed in patients with early cervical cancer. - LRH reduces the morbidity associated with abdominal or transvaginal radical hysterectomy.
Zakashansky K	2007	- It is feasible to incorporate TLRH training into the surgical curriculum of gynecologic oncology fellows without increasing perioperative morbidity. - Standardization of TLRH technique and consistent guidance by experienced faculty is imperative.
Ghezzi F	2007	- Laparoscopically managed patients with cervical cancer undergo a similar extent of surgery as those treated with the traditional ORH, as judged by objective pathologic criteria.
Frumovitz M	2007	- TLRH reduces operative blood loss, postoperative infectious morbidity and postoperative length of stay. - TLRH did not sacrifice the size of radical hysterectomy specimen margins. - TLRH is associated with increased operative time.
Li G	2007	- LRH and pelvic lymphadenectomy has similar efficacy and recurrence rates to ORH and pelvic lymphadenectomy. - LRH is a safe and effective alternative to ORH for stage Ib–IIa cervical cancer. - LRH should be used if the surgeon is sufficiently trained. - Its clinical value should be confirmed by multicenter randomized clinical trials.
Boggess JF	2008	- RRH type III with pelvic node dissection is feasible and may be preferable over ORH in patients with early-stage cervical cancer. - Further study will determine procedure generalizability and long-term oncologic outcomes.
Chen Y	2008	- LRH is a routine, effective treatment for patients with Ia2–IIb cervical carcinoma. - With more experience, it is envisaged that stage IIb patients can be managed safely, offering all benefits of minimal surgery to patients.
Ko EM	2008	- RRH results in lower blood loss and shorter length of stay compared to ORH. - Intraoperative and postoperative complication rates are comparable. - Promising new surgical technique that deserves further study.
Nezhat FR	2008	- RRH appears to be equivalent to TLRH with respect to operative time, blood loss, hospital stay and oncological outcome. - The intuitive nature of the robotic approach, magnification, dexterity and flexibility combined with significant reduction in surgeon fatigue offered by the robotic system will allow more surgeons to use a minimally invasive approach to radical hysterectomy.
Magrina JF	2008	- Laparoscopy and robotics are preferable to laparotomy for patients requiring radical hysterectomy. - Operating times for robotics and laparotomy were similar, and significantly shorter as compared to laparoscopy. - Blood loss, rate of blood loss and length of hospital stay were similar for laparoscopy and robotics, and significantly reduced as compared to laparotomy.
Díaz-Feijoo B	2008	- Sentinel lymph node identification and radical hysterectomy in the initial treatment of early-stage cervical cancer can be performed safely by laparoscopy with lower morbidity and overall survival, and with recurrence-free survival similar to standard laparotomy.
Pellegrino A	2009	- TLRH, in experienced hands, has to be considered an adequate and feasible surgical technique. - Considering historical data, the oncological outcome can be considered comparable to patients treated with laparotomy, as the relapse rate in our population was 11% and the overall survival good.
Estape R	2009	- RRH hysterectomy with pelvic and para-aortic lymphadenectomy is feasible and may be preferable over laparoscopic or radical abdominal hysterectomy.
Sobiczewski P	2009	- TLRH may be an option in early cervical cancer. - The intraperitoneal spread in 2 patients compels a search for possible risk factors in patients managed by laparoscopy.
Lowe MP	2009	- No major operative complications occurred with RRH. - RRH was associated with a significant reduction in blood loss and hospital stay. - Improved nodal yields, fewer operative complications and less pain was observed with the robotic approach. - RRH appears safe and feasible and further investigation is warranted in a prospective fashion.
Maggioni A	2009	- RRH is safe and feasible. - A comparison of oncologic outcomes and cost–benefit analysis are still needed and it has to be carefully evaluated in the future.
Malzoni M	2009	- TLRH is a safe and effective therapeutic procedure for management of early-stage cervical cancer with far lower morbidity than reported for the open approach. - TLRH is characterized by far less blood loss and shorter postoperative hospitalization time. - Multicenter randomized clinical trials with longer follow-up are necessary to evaluate the overall oncologic outcomes of this procedure.
Cantrell LA	2010	- Surgical outcomes of RRH and pelvic lymphadenectomy were comparable to that of laparoscopic approach, with significantly less blood loss and early postoperative complications.
Lee CL	2010	- The laparoscopic approach has favorable long-term survival outcomes and perioperative morbidity. - With the advantage of minimal invasiveness, laparoscopic treatment by experienced surgeons is an ideal alternative for early-stage cervical cancer.
Geisler JP	2010	- Radical surgery for cervical cancer can be accomplished using the da Vinci surgical system (Intuitive Surgical, Sunnyvale, Calif) with acceptable blood loss, operating time, parametrial margins, and nodal yield. - Future studies need to address long-term outcomes.
Gocmen A	2010	- Robotic surgery was superior to laparotomy in terms of duration of hospital stay, estimated amount of blood loss and number of complications. - Operation duration was longer with robotic surgery compared with laparotomy, and rate of complications was higher with laparotomy.
Halliday D	2010	- Whereas robotics takes longer to perform than traditional laparotomy, it provides the patient with a shorter hospital stay, less need for pain medications and reduced peri-operative morbidity. - In addition, real average hospital costs tend to be lower.
Nam EJ	2010	- RRH and pelvic lymphadenectomy using 3 robotic arms is feasible and preferable over ORH for the treatment of cervical cancer patients. - Prospective randomized trials should be completed to confirm the potential benefits associated with robotic radical hysterectomy.
Schreuder H	2010	- RRH is feasible and of benefit to patients with early-stage cervical cancer via a reduction in blood loss and reduced hospital stay. - Introduction of this new technique requires a learning curve of less than 15 cases that will reduce the operating time to a level comparable to open surgery.
Mehra G	2010	- Vaginal radical hysterectomy with laparoscopic pelvic lymphadenectomy is feasible and safe with regard to mortality, and has low morbidity.
Sert MB	2011	- RRH and pelvic lymph node dissection is feasible and more precise because the instruments provide better flexibility and 3D vision. - Patients subjected RRH should be followed carefully and RRH would be encouraged as part of a protocol until the long-term oncological outcome data are available.
Lee EJ	2011	- LRH is a useful alternative to ORH for the management of early-stage cervical cancer. - The benefits of LRH include reduced blood loss, fewer transfusions and shorter hospital stay, with comparable oncologic outcome.
Soliman PT	2011	- Minimally invasive surgery has made a significant impact on patients undergoing radical hysterectomy including decrease in blood loss and transfusion rates. - Operative times were significantly longer compared to ORH. - The robotic approach may have the added benefit of even shorter length of stay compared to traditional laparoscopy.
Taylor SE	2011	- LRH is a feasible alternative to laparotomy for early-stage cervical cancer. - Similar surgical outcomes are achieved with significantly less morbidity.
Tinelli R	2011	- RRH can be considered a safe and effective therapeutic procedure for managing early-stage cervical cancer without significant differences, when compared with laparoscopic radical hysterectomy, in terms of the recurrence rate and intraoperative and postoperative complications. - Multicenter randomized clinical trials with longer follow-up are necessary to evaluate the overall oncologic outcomes of this procedure.
Gortchev G	2012	- RRH has been established to be a safe procedure with proven advantages in regard to operative time and hospital stay. - The absence of significant differences in DFS and OS is a substantial reason to continue, from an oncologic point of view, the application of this method in patients with T1в1 cervical cancer.
Park JY	2012	- LRH was a preferred alternative to ORH in the present cohort of obese women with early-stage cervical cancer because it is associated with a more favorable surgical outcome without compromising survival outcomes.
Lanowska M	2012	- LARVH is a feasible and oncologically safe surgical option for patients with early-stage cervical cancer. - The complication rate is lowered in LARVH by the combination of the laparoscopic and vaginal approach.
Stanciu P	2013	- LRH is safe and has lower operative complication rates than ORH.
Park JY	2013	- LRH has similar therapeutic efficacy to ORH in patients with bulky early-stage cervical cancer. - LRH has more favorable surgical outcomes. - LRH is not only a reasonable alternative to ORH but also the preferred surgical procedure for these patients.
Lim YK	2013	- With appropriate patient selection and increased experience, TLRH can be a safe and effective procedure for management of early cervical cancer in Singapore.
Campos LS	2013	- LRH provided lower pain scores after 36 h of observation. - The perioperative and serious postoperative complication ratios were comparable with ORH.
Gemer O	2013	- Using a preoperative triage algorithm, patients with early small lesions, no LVSI and no nodal involvement may be spared radical surgical procedures and parametrectomy. Further prospective data are urgently needed.
Toptas T	2014	- TLRH and ORH have similar survival outcomes in patients with early-stage cervical cancer.
Hoogendam JP	2014	- The recurrence, survival and long-term complication rates of RRH for early-stage cervical cancer in this cohort are reassuring concerning its continued clinical use.
Bogani G	2014	- Laparoscopy ensures the same results as open surgery insofar as radicality and long-term survival. - Use of the laparoscopic approach is associated with improved short-term results, minimizing the occurrence of severe postoperative complications.
Kong TW	2014	- LRH might be a feasible therapeutic procedure for the management of FIGO stage IB and IIA cervical cancer with tumor diameter of 3 cm or greater. - Further randomized studies that could support this approach are necessary to evaluate long-term clinical outcome.
Segaert A	2015	- This series confirms the feasibility and safety of RRH not only in cervical cancer stage IA to IB1, but also after neoadjuvant chemotherapy in stage IB2 to IIB.
Garabedian C	2015	- The laparoscopic approach was favorable for both perioperative and postoperative morbidity. - With the advantage of minimal invasiveness, laparoscopic treatment by experienced surgeons is an alternative for early-stage cervical cancer with acceptable long-term survival outcomes. - Mini-invasive surgery could be the standard in early-stage cervical cancer.
Pessini S	2015	- The 2- and 5-year survival of cervical cancer patients treated by LARVH were similar to patients treated by ORH. - The average of lymph nodes by laparoscopy was greater than laparotomy, without significant difference.
Xiao M	2015	- Total laparoscopic procedure is a surgically and oncologically safe and reliable alternative to laparotomic procedure in the treatment for cervical cancer.
Yang L	2015	- LRH is adequate, safe and feasible for women with cervical cancer, and it can be routinely used for treatment of early-stage tumors as a primary modality.
Ditto A	2015	- LRH is a safe procedure and upholds the results of ORH, reducing invasiveness of open surgical operations. - Further large prospective investigations are warranted.
Kong TW	2016	- Total laparoscopic/robotic intracorporeal colpotomy under CO_2_ pneumoperitoneum may carry risk of positive vaginal cuff margin, as well as intraperitoneal tumor spread in patients with early-stage cervical cancer treated with laparoscopic radical hysterectomy/robotic radical hysterectomy.
Laterza RM	2016	- Early-stage cervical cancer can be treated with LRH with similar recurrence rates and patterns in comparison with ORH, reassuring its continuing clinical use.
Wang W	2016	- LRH was associated with less operating time, blood loss, postoperative complications, and earlier recovery. - LRH is an oncologically safe alternative to ORH.
Park JY	2016	- LRH has comparable survival outcomes with ORH and did not affect the pattern of recurrence in early-stage adenocarcinoma of the uterine cervix. - The surgical outcomes were more favorable than ORH.
Zanagnolo V	2016	- RRH is safe and feasible and is associated with improved clinical outcomes. - Although longer follow-up is needed, early data show equivalent oncologic outcomes compared with other surgical modalities.
Mendivil AA	2016	- Irrespective of operative approach, patients who underwent radical hysterectomy for early-stage cervical cancer attained similar 5-year disease-free and overall survival outcomes.
Diver E	2017	- Minimally invasive radical hysterectomy does not compromise patient outcomes, including overall survival, rate of recurrence, and the frequency of pelvic lymph node dissection or positivity. - Morbidity was decreased in the minimally invasive group, including decreased estimated blood loss, fewer blood transfusions and shorter hospital stay.
Zhang S	2017	- LARVH is a suitable alternative to ORH for early-stage cervical cancer, which shows less blood loss, shorter catheterized and hospital stay, and similar survival outcomes.
Pellegrino A	2017	- RRH is safe and feasible and is associated with improved intraoperative results and clinical oncological outcomes.

LRH = laparoscopic radical hysterectomy, LARVH = laparoscopic-assisted vaginal radical hysterectomy, TLRH = total laparoscopic radical hysterectomy, RRH = robotic radical hysterectomy, ORH = open radical hysterectomy.

**Table 4:** Literature published after 2018—main conclusions of these studies were cited.

First Author	Year of Publication	Main Conclusions
Kimmig R	2018	- Histological data on tumor size were missing in a third of abdominal hysterectomy cases in LACC trial. - The incidence of lymphovascular space invasion was missing in 5% of cases. - Data on parametrial and vaginal involvement were missing in 7% and 10% of cases, respectively, in the abdominal arm of the trial. - The operative outcomes from the LACC study need to be known. - Patients should be informed of all the known data and decide on the treatment strategy.
Kanao H	2019	- The no-look no-touch technique may be a useful surgical procedure to reduce recurrence risk via preventing intraoperative tumor spillage during TLRH for early-stage cervical cancer.
Hillemanns P	2019	- Even surgeons with extensive experience of performing LRH or RRH to treat early-stage (up to FIGO stage IB1) cervical cancer should inform every patient in detail prior to surgery about the provisional results of LACC trial.
Chao X	2019	- In this phase III multicenter randomized controlled study, study centers and individual surgeons will be integrated as important influencing factors in survival outcomes in patients with early-stage cervical cancer receiving different surgical approaches (MIS vs. ORH).
Falconer H	2019	- Primary endpoint: Recurrence-free survival at 5 years between women who underwent robot-assisted laparoscopic surgery versus laparotomy for early-stage cervical cancer. - Trial launch was estimated to be May 2019 and the trial is estimated to close in May 2027 with presentation of data shortly thereafter.
Nitecki R	2020	- Minimally invasive radical hysterectomy was associated with an elevated risk of recurrence and death compared with open surgery.
Kampers J	2021	- DFS and OS in laparoscopy appear to be dependent on surgical technique. - Protective operating techniques in laparoscopy result in improved minimal invasive survival.
Liang S	2021	- MIS should not be abandoned. - Another prospective randomized trial is need, including a single pathologist review, perioperative MRI, parametrical measurements and quality indicators of radical hysterectomy.
Lewicki PJ	2021	- Substantial reduction in the use of minimally invasive surgery for cervical cancer after publication of the results of the LACC trial. - The use of this approach among nonacademic providers suggests an opportunity to improve outcomes.
Benny Brandt	2022	- Modification of surgical technique and maintaining proper oncologic surgical principles are key for MIS to remain a viable option. - Tumor manipulation and contamination should be avoided. - Transcervical uterine manipulators should not be used.
Fusegi A	2022	- Avoiding cancer cell spillage during MIS radical hysterectomy may ensure an equivalent oncologic outcome, comparable to that of ORH.
Li RZ	2022	- MIS radical hysterectomy without using a uterine manipulator resulted in an inferior recurrence-free survival compared with ORH in the treatment of women with early-stage cervical cancer.
Bogani G	2022	- A minimally invasive approach should only be offered in the context of controlled trials. - Further evidence from well-designed retrospective studies is warranted to improve knowledge on the treatment of early-stage cervical cancer.
Giannini, A	2022	- No significant differences in FIGO IB–IIA cervical adenocarcinoma between open surgery and minimally invasive surgery.
Wu X	2022	- The primary endpoint will be 5-year progression-free survival, and secondary endpoints include 5-year overall survival, recurrence, and quality of life measurements.
Giacomo Corrado	2023	- MIS, in FIGO stage IB1–IB2 cervical cancer, is not associated with different relapse patterns compared to ORH, nor with a higher risk of distant metastasis and finally, no significant difference in terms of DFS and OS was detected.
Pedro T Ramirez	2024	- Given higher recurrence rate and worse overall survival with minimally invasive surgery, an open approach should be standard of care.
Song YL	2024	- MIS radical hysterectomy with protective colpotomy for treatment of early-stage cervical cancer had similar DFS and OS compared to ORH.

LRH = laparoscopic radical hysterectomy, LARVH = laparoscopic-assisted vaginal radical hysterectomy, TLRH = total laparoscopic radical hysterectomy, RRH = robotic radical hysterectomy, ORH = open radical hysterectomy, DFS = disease-free survival, OS = overall survival, MIS = minimally invasive surgery.

## Data Availability

The data generated in the present study may be requested from the corresponding author.

## Abbreviations

HPV	Human papillomavirus
FIGO	International Federation of Gynecology and Obstetrics
LACC	Laparoscopic approach to cervical cancer
MIS	Minimally invasive surgery
LRH	Laparoscopic radical hysterectomy
LARVH	Laparoscopic-assisted vaginal radical hysterectomy
TLRH	Total laparoscopic radical hysterectomy
RRH	Robotic radical hysterectomy
ORH	Open radical hysterectomy
DFS	Disease-free survival
OS	Overall survival
NCCN	National Comprehensive Cancer Network
ESMO	European Society for Medical Oncology
ESGO	European Society of Gynaecological Oncology
ESTRO	European Society for Radiotherapy and Oncology
ESP	European Society of Pathology
MRI	Magnetic resonance imaging
CT	Computed tomography
PET-CT	Positron emission–computed tomography scan
18-FDG	18-fludeoxyglucose
